# A transgenic mouse model of human T cell leukemia virus type 1-associated diseases

**DOI:** 10.3389/fmicb.2013.00049

**Published:** 2013-03-08

**Authors:** Takeo Ohsugi

**Affiliations:** Division of Microbiology and Genetics, Institute of Resource Development and Analysis, Kumamoto UniversityKumamoto, Japan

**Keywords:** animal model, ATLL, HTLV-1, Tax, transgenic mice

## Abstract

Human T cell leukemia virus type 1 (HTLV-1) is the etiological agent of adult T cell leukemia/lymphoma (ATLL) and several inflammatory diseases. Tax, the protein encoded by HTLV-1, may be responsible for the development of the diseases caused by this virus. To investigate the pathogenic role of Tax, several transgenic mouse strains expressing Tax have been developed in recent years. These mice develop various tumors including large granular lymphocytic leukemia, as well as inflammatory diseases such as arthritis. These results suggest that Tax expression alone is sufficient to cause both malignant neoplastic diseases and inflammatory diseases. However, until recently, there were no *tax* transgenic mice that develop T cell leukemia and lymphoma resembling ATLL. The first successful induction of leukemia in T cells was pre-T cell leukemia generated in transgenic mice in which a mouse lymphocyte-specific protein tyrosine kinase p56^*lck*^ (*lck*)-proximal promoter was used to express the *tax* gene in immature T cells. Subsequently, transgenic mice were established in which the *lck*-distal promoter was used to express Tax in mature T cells; these mice developed mature T cell leukemia and lymphoma that more closely resembled ATLL than did earlier mouse models.

## INTRODUCTION

Human T cell leukemia virus type 1 (HTLV-1) was the first human retrovirus to be isolated (Poiesz et al., 1980). It is estimated that 10–20 million people worldwide are infected with HTLV-1, which is endemic in southwestern Japan, the Caribbean Islands, South America, and Africa. Infection with HTLV-1 can result in an aggressive malignancy known as adult T cell leukemia/lymphoma (ATLL) or in inflammatory diseases, such as HTLV-1-associated myelopathy/tropical spastic paraparesis (HAM/TSP), after a prolonged period of latency often lasting between 20 and 50 years (Watanabe, 1997). The lifetime incidence of ATLL among carriers of HTLV-1 was estimated to be 1–5%, whereas that of HAM/TSP was 0.3–4.0% (Verdonck et al., 2007). The lifetime incidence of HTLV-1-associated diseases in general, including ATLL, HAM/TSP, and other inflammatory diseases, such as uveitis, polymyositis, arthropathy, and infective dermatitis, may be close to 10% (Verdonck et al., 2007). However, the reasons why HTLV-1-infected individuals develop different types of diseases and the mechanisms through which HTLV-1 causes these diseases remain unclear. To address these questions, an appropriate animal model is required.

## HTLV-1 GENOME

The HTLV-1 genome consists of a diploid plus-strand RNA. Like other retroviruses, the integrated HTLV-1 proviral genome contains long-terminal-repeat (LTR) regions flanking the genes encoding the major structural proteins, *gag*, *pol*, and *env*. The genome also has an extra sequence designated *pX*. The *pX* region has four partially overlapping open reading frames designated I, II, III, and IV, which encode the proteins p12, p13, and p30, Rex, and Tax, respectively (Grassmann et al., 2005; Matsuoka and Jeang, 2007). Tax and Rex act in combination to regulate HTLV-1 gene expression and replication in both positive and negative pathways (Yoshida, 2005). p12 is thought to facilitate persistent viral infection (Albrecht et al., 2000). p30 attenuates HTLV-1 transcription by suppressing Tax protein synthesis (Nicot et al., 2004). The role of p13 is currently unclear. The HTLV-1 minus-strand RNA encodes a basic leucine zipper factor (HBZ) and the protein is synthesized in an antisense fashion from the 3′ LTR (Larocca et al., 1989; Gaudray et al., 2002). HBZ inhibits Tax-mediated transactivation of viral transcription (Arnold et al., 2006; Lemasson et al., 2007; Clerc et al., 2008). However, several researchers have reported that HBZ mRNA, but not HBZ protein, could induce T cell proliferation and to promote cell survival (Satou et al., 2006; Arnold et al., 2008). At present, the role of HBZ in HTLV-1 infection is controversial. More recently, Satou et al. (2011) created *hbz* transgenic mice and reported that more than one-third of these mice developed T cell lymphoma after a long latent period.

The transcription activator protein, Tax, is one of the regulatory proteins encoded by the *pX* region that has been extensively studied *in vitro*. Tax is a 40 kDa phosphoprotein that is essential for both viral replication and cellular transformation (Yoshida, 2001; Jeang et al., 2004). Transactivation of Tax is thought to initiate the processes that lead to ATLL or inflammatory diseases (Sun and Yamaoka, 2005; Matsuoka and Jeang, 2007; Currer et al., 2012; Yamagishi and Watanabe, 2012).

## *tax* TRANSGENIC MICE

One of the best ways to investigate the oncogenic role(s) of *tax in vivo* is to generate a transgenic mouse model expressing HTLV-1 Tax (**Table 1**). The first HTLV-1 *tax* transgenic mice, in which Tax was expressed under the control of the HTLV-1 LTR, developed thymic involution, neurofibroma, and early death (Hinrichs et al., 1987; Nerenberg et al., 1987). Studies of these mice indicated that Tax expression alone was sufficient to induce tumorigenesis in transgenic mice. Iwakura et al. subsequently reported a very high incidence of inflammatory arthritis in transgenic mice carrying the HTLV-1 *env*–*pX* region (*pX* transgenic mice) or *tax* with the HTLV-1 LTR promoter (Iwakura et al., 1991; Habu et al., 1999). Arthropathy develops in *pX* transgenic mice as early as 4 weeks of age, and inflammatory arthropathy was also reported in another *tax* transgenic mouse model (Saggioro et al., 1997). These reports suggest that Tax expression induces inflammatory diseases in mice. Other transgenic mice were reported to develop Sjögren’s-like syndrome (Green et al., 1989) and skeletal abnormalities (Ruddle et al., 1993).

**Table 1:** Representative *tax* transgenic mouse models.

Promoter	Gene	Diseases	ATLL-like	HAM/TSP-like	Reference
HTLV-1 LTR	*tax*	Thymic involution Neurofibroma Early death	None	None	Hinrichs et al. (1987) Nerenberg et al. (1987)
				
HTLV-1 LTR	*tax*	Sjögren-like syndrome (exocrinopathy)	None	None	Green et al. (1989)
HTLV-1 LTR	*env-pX*	Arthritis	None	None	Iwakura et al. (1991)
HTLV-1 LTR	*tax*	Arthritis	None	None	Habu et al. (1999)
CD4	*tax*	Arthritis	None	None	
HTLV-1 LTR	*tax*	Skeletal abnormalities	None	None	Ruddle et al. (1993)
Granzyme B	*tax*	Granular lymphocytic leukemia	Leukemia/lymphoma	None	Grossman et al. (1995)
Metallothionein	*tax*	Arthropathy	None	None	Saggioro et al. (1997)
*lck*-proximal	*tax*	CD4^-^CD8^-^pre-T-cell leukemia	Leukemia/lymphoma	None	Hasegawa et al. (2006)
*lck*-distal	*tax*	CD4^+^CD8^+^, and CD4^+^CD8^+^ T-cell leukemia Arthropathy Histiocytic sarcoma (spinal cord)	Leukemia/lymphoma	Symmetrical paraparesis of the hind limbs	Ohsugi et al. (2007)

However, none of these transgenic mouse models developed leukemia and lymphoma. The HTLV-1 LTR was used to regulate *tax* expression in these models. Other promoters were used in transgenic constructs to restrict *tax* expression to the lymphoid compartment and establish a better model of ATLL-like malignancies. Grossman et al. (1995) used the granzyme B promoter to drive *tax* expression in the mature T cell compartment. Those mice developed large granular lymphocytic leukemia, demonstrating that Tax expression alone in the lymphocyte compartment is sufficient for the development of leukemia.

## T CELL LEUKEMIA IN *tax* TRANSGENIC MICE

Tax expression in transgenic mice caused large granular lymphocytic leukemia, but none of the transgenic mice developed T cell leukemia and lymphoma resembling ATLL. Recently, Hasegawa et al. (2006) established transgenic mice in which *tax* expression was restricted to thymocytes by using the lymphocyte-specific protein tyrosine kinase p56^*lck*^ (*lck*)-proximal promoter. These mice developed pre-T cell (CD4^-^CD8^-^CD44^+^CD25^+^) leukemia and lymphoma. Histological analysis showed diffuse, large-cell lymphomas involving the spleen, lymph nodes, liver, thymus, bone marrow, kidney, lung, meninges, and skin. The histopathological findings were identical to those observed in ATLL patients. The mice were functionally immunocompromised and developed opportunistic infections, which are also characteristics of ATLL. The leukemic cells were transplantable to severe combined immunodeficient mice. These transgenic mice demonstrated that Tax expression in the lymphocyte compartment is sufficient for the development of T cell leukemia and lymphoma. One major difference between these mice and humans with the disease is in the phenotype of the tumor cells, as the most common phenotype in ATLL in humans is CD4^+^ mature T cells (Watanabe et al., 1997; Matsuoka and Jeang, 2007; Verdonck et al., 2007).

We created a transgenic mouse model of HTLV-1 using the distal promoter of *lck *to express *tax* in mature T cells (Ohsugi et al., 2007). The expression of the *lck* gene is regulated by two distinct promoter elements, a proximal and a distal promoter (Voronova et al., 1987; Perlmutter et al., 1988; Takadera et al., 1989). The *lck*-proximal promoter is most active in immature thymocytes, whereas the activity of the distal promoter is higher in mature thymocytes and peripheral T lymphocytes (Reynolds et al., 1990; Wildin et al., 1991; Allen et al., 1992). Tax mRNA expression in various organs of the transgenic mice was examined by quantitative real-time RT-PCR. The thymus and spleen strongly expressed Tax mRNA. Over 2 years, 28.1% of the *tax* transgenic mice developed mature T cell leukemia/lymphoma compared with just 1% of non-transgenic littermates.

## PHENOTYPE OF T CELL LEUKEMIA IN *tax* TRANSGENIC MICE

**FIGURE 1:**
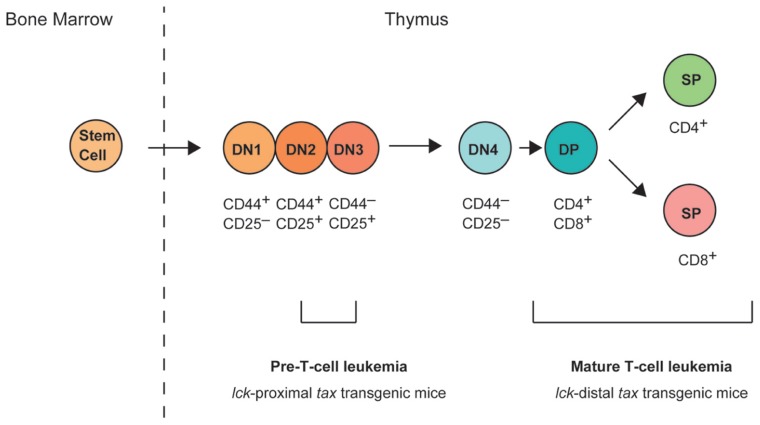
**T cell development in the thymus**. Hematopoietic stem cells arise in the bone marrow and migrate to the thymus. Early committed T cells do not express CD4 or CD8, and are referred to as double-negative (DN; i.e., CD4^-^ or CD8^-^) thymocytes. DN thymocytes can be further subdivided into four stages of differentiation (DN1, CD44^+^CD25^-^; DN2, CD44^+^CD25^+^; DN3, CD44^-^CD25^+^; and DN4, CD44^-^CD25^-^). At the DN3 stage, DN cells express a pre-T cell receptor (TCR) and its surface expression triggers the upregulation of CD4 and CD8 expression on the cell surface, and progression to the CD4^+^CD8^+^ double-positive (DP) stage. DP cells then mature into either CD4^+^ or CD8^+^ single-positive (SP) T cells. *lck*-proximal *tax* transgenic mice develop pre-T-cell leukemia originating from DN2/3 stage cells, whereas *lck*-distal *tax* transgenic mice develop mature leukemia originating after the progression to DP stage cells.

The final cell specification to the T cell lineage takes place within the thymus. An overview of thymic T cell development is illustrated in **Figure 1**, and involves a series of distinct stages that can be defined by the expression of cell-surface markers. Early T cells are CD4^-^CD8^-^ double-negative (DN) and can be further subdivided into DN1 (CD44^+^CD25^-^), DN2 (CD44^+^CD25^+^), DN3 (CD44^-^CD25^+^), and DN4 (CD44^-^CD25^-^) stages based on their expression of CD25 and CD44 (Koch and Radtke, 2011). The productive rearrangement of the T cell receptor (TCR) β chain locus occurs during the DN3 to DN4 transition, and leads to the expression of the pre-TCR. Only cells expressing functional pre-TCR proliferate and differentiate into CD4^+^CD8^+^ double-positive (DP) cells. However, most DP cells die through negative selection or neglect because their TCRs have too high or too low affinity for peptide–major histocompatibility complex molecule complexes. The cells that mature successfully migrate to the periphery as functional CD4^+^CD8^-^ or CD4^-^CD8^+^ single-positive T cells.

The phenotype of T cell leukemia in *lck*-proximal *tax* transgenic mice was CD4^-^CD8^-^, but CD44^+^, CD25^+^, and CD117^-^ (also known as c-kit^-^; Hasegawa et al., 2006). These results suggest that the malignant leukemic cells were derived from DN2/3 stage cells in the thymus. Transgenes controlled by the *lck*-proximal promoter are expressed very early in T cell development and can be detected in DN1 cells, although their expression in the early progenitors is not homogeneous until the DN3/4 stage (Cleverley et al., 1999; Buckland et al., 2000). Various studies have suggested that the Lck tyrosine kinase plays an important role in thymocyte development as a signaling molecule downstream from pre-TCR (Mombaerts et al., 1994; Wallace et al., 1995; van Oers et al., 1996; Fehling et al., 1997), while pre-TCR was first detected in the CD44^-^CD25^+^ DN3 subset (Saint-Ruf et al., 1994; Bruno et al., 1995). A green fluorescent protein (GFP) transgenic mouse was created in which *gfp* expression was under the control of the proximal promoter of *lck*, and the earliest GFP-positive cells were found among the CD44^+^CD25^-^ DN1 stage cells (Shimizu et al., 2001). Interestingly, pre-T cell leukemia in *lck*-proximal *tax* transgenic mice was probably derived from DN2/3 stage cells, even though the *lck*-proximal promoter is active in DN1–DN4 stage cells.

The phenotype of T cell leukemia in *lck*-distal *tax* transgenic mice displayed CD4^+^, CD8^+^, or CD4^+^CD8^+^ (DP) T cells. Overall, 60% of the leukemic cells were CD8^+^CD25^-^ T cells, 25% were CD4^+^CD25^-^ T cells, and 15% were CD4^+^CD8^+^ T cells (Ohsugi et al., 2007). As the Tax expression level did not vary in these cell populations, it remains unclear why CD8^+^ T cells comprised approximately 60% of the total mature T cell leukemia cells in the *lck*-distal *tax* transgenic mice.

## TAX-RELATED DISEASES IN *tax* TRANSGENIC MICE

### ARTHROPATHY

*tax* transgenic mice develop an inflammatory arthropathy (Iwakura et al., 1991; Saggioro et al., 1997) that is pathologically similar to human rheumatoid arthritis and to mouse models of rheumatoid arthritis, with synovial proliferation and the expression of rheumatoid factor (Lee and Weinblatt, 2001; Luross and Williams, 2001; Firestein, 2003). By 24 months of age, our established *lck*-distal *tax* transgenic mice without leukemia developed severe arthropathy, with a cumulative incidence of 22.8% (Ohsugi and Kumasaka, 2011), but no arthropathic mouse was reported among the *lck*-proximal *tax* transgenic mice. The *lck*-distal *tax* transgenic mice with arthropathy differ in several aspects from other transgenic mice. They develop arthropathy after a prolonged latency period of at least 9 months, whereas the arthropathy that develops in the *pX* transgenic mice occurs as early as 4 weeks of age. At 3 months of age, 60% (BALB/c background), 25% (C3H/He background), and 0% (C57BL/6 background) of *pX* transgenic mice displayed arthropathy (Iwakura et al., 1998). The genetic background of our established *lck*-distal *tax* transgenic mice was the F1 generation of a BDF1 (DBA/2×C57BL/6) cross with C57BL/6. We attempted to generate *lck*-distal *tax* transgenic mice with the BALB/c background (backcross generation 8: N8), but did not observe a high incidence of arthropathy by 24 months of age. The expression of the cytokines interleukin-1β (IL-1β), IL-6, and macrophage migration inhibitory factor (MIF) was markedly enhanced in the joints of the *lck*-distal *tax* transgenic mice, but the expression of tumor necrosis factor-α (TNF-α) was not elevated. Ashino et al. (2007) also found that serum IL-1β and IL-6 concentrations were significantly higher in *pX* transgenic mice than those in non-transgenic or non-arthritic *pX* transgenic mice. Consistent with our arthropathic mice, their serum TNF-α concentrations were low, with no significant differences between the groups (Ashino et al., 2007). IL-6 is a key proinflammatory cytokine that is abundant in the synovium and synovial tissues of patients with rheumatoid arthritis (Okamoto et al., 1997). Taken together, these data suggest that proinflammatory cytokines, other than TNF-α, are important in the development of the inflammatory arthropathy associated with Tax expression.

### HAM/TSP-LIKE DISEASE

Eight out of 297 *lck*-distal *tax* transgenic mice developed HAM/TSP-like disease with symmetrical paraparesis of the hind limbs, whereas these symptoms were absent in their non-transgenic littermates and in other mouse strains at our animal facilities (Ohsugi et al., in press). The *tax* transgenic mice with HAM/TSP-like disease had spinal cord lesions in the lumbar vertebrae that were caused by the infiltration of bone marrow-derived histiocytic sarcoma cells. Mice with HAM/TSP-like disease also displayed abnormal expression of cytokines and chemokines, including TNF-α and IL-6. Constitutive exposure to high levels of proinflammatory cytokines is thought to be protumorigenic (Balkwill, 2009; Grivennikov et al., 2009). Therefore, we speculated that *tax*-expressing T cells stimulate the proliferation of histiocytic cells in bone marrow through the activities of cytokines or chemokines. The transformed histiocytic cells may then predominantly invade the lumbar spinal cord (Ohsugi et al., in press).

To my knowledge, there have been no reports of spontaneous symmetrical paraparesis caused by histiocytic sarcoma in mice. However, the lesions in patients with HAM/TSP show marked T cell infiltration, and the disease is associated with an inflammatory state (Sakai et al., 2001; Kubota et al., 2002; Goon et al., 2003; Muraro et al., 2003). Therefore, it is important to note that the etiology of HAM/TSP-like disease in *lck*-distal *tax* transgenic mice differs substantially from that of HAM/TSP in humans. Nevertheless, the present results indicate that the relationship between HTLV-1 infection and histiocytic disorders should be the focus of future studies. In particular, those studies should examine whether the cytokines and chemokines secreted from HTLV-1-infected T cells induce the growth or oncogenic transformation of histiocytic cells in humans. A recent paper proposed that, in a murine model of multiple sclerosis, there is a gateway through which immune cells can enter the central nervous system (Arima et al., 2012). The authors described an entry site by the dorsal blood vessels of the fifth lumbar cord through which immune cells can enter the central nervous system. Histiocytic sarcoma grew predominantly in the spinal cord of the fifth to sixth lumbar vertebrae in HAM/TSP-like mice. Further studies are required to confirm that histiocytic sarcoma cells can access the central nervous system via the spinal cord at the fifth lumbar vertebra. Such studies may clarify the mechanisms underlying the movement of Tax-positive T cells into the central nervous system in humans with HAM/TSP.

## CONCLUSION

Several strains of HTLV-1 *tax* transgenic mice have been developed over recent years. Studies in these mice have shown that Tax expression alone is sufficient to cause both malignant neoplastic diseases, including T cell leukemia and lymphoma, and inflammatory diseases, such as arthropathy. These mice will be widely used to study the pathogenesis of HTLV-1 and to evaluate new anticancer and anti-inflammatory agents for HTLV-1-related diseases. However, until now, none of the *tax* transgenic mice developed HAM/TSP-like disease, a systemic immune-mediated inflammatory disease that resembles the disease in humans. Further studies are required to establish a transgenic mouse model of HAM/TSP with the selection of appropriate promoters.

## Conflict of Interest Statement

The author declares that the research was conducted in the absence of any commercial or financial relationships that could be construed as a potential conflict of interest.
